# Minerals Determined a Special Ecological Niche and Selectively Enriched Microbial Species from Bulk Water Communities in Hot Springs

**DOI:** 10.3390/microorganisms9051020

**Published:** 2021-05-10

**Authors:** Fangru Li, Shang Wang, Qing He, Wenhui Zhang, Dongyi Guo, Yidi Zhang, Wanming Hai, Yuxuan Sun, Hailiang Dong, Weiguo Hou

**Affiliations:** 1 State Key Laboratory of Biogeology and Environmental Geology, China University of Geosciences, Beijing 100083, China; fangruli@cugb.edu.cn (F.L.); 2157190005@cugb.edu.cn (W.Z.); dongyiguo@cugb.edu.cn (D.G.); 2157180007@cugb.edu.cn (Y.Z.); 2157180004@cugb.edu.cn (W.H.); 2157180006@cugb.edu.cn (Y.S.); dongh@cugb.edu.cn (H.D.); 2 CAS Key Laboratory of Environmental Biotechnology, Research Center for Eco-Environmental Sciences, Chinese Academy of Sciences, Beijing 100085, China; shangwang@rcees.ac.cn (S.W.); qinghe_st@rcees.ac.cn (Q.H.)

**Keywords:** network analysis, niche partitioning, carbonate minerals, silicate minerals, selective effect, microbial species

## Abstract

Minerals provide physical niches and supply nutrients or serve as electron donors/acceptors for microorganism survival and growth, and thus minerals and microbes co-evolved. Yet, little is known about how sediment minerals impact microbial community assembly in hot springs and to what extent mineralogical composition influences microbial community composition and diversity. Here the influences of minerals on thermophiles in Tengchong hot springs were revealed by network analysis of field samples, as well as in-situ microcosm experiments with minerals. A molecular ecological network was constructed based on high throughput sequencing data of 16S rRNA gene, with a combination of water geochemistry and sedimentary mineralogical compositions. Six modules were identified and this highly modular network structure represents the microbial preference to different abiotic factors, consequently resulting in niche partitioning in sedimentary communities in hot springs. Diverse mineralogical compositions generated special niches for microbial species. Subsequently, the in-situ microcosm experiments with four minerals (aragonite, albite, K-feldspar, and quartz) and spring water were conducted in a silicate-hosted alkaline spring (i.e., Gmq) and a carbonate-hosted neutral hot spring (i.e., Gxs) for 70 days. Different microbial preferences were observed among different mineral types (carbonate versus silicate). Aragonite microcosms in Gmq spring enriched archaeal genera Sulfophobococcus and Aeropyrum within the order Desulfurococcales by comparison with both in-situ water and silicate microcosms. Sulfophobococcus was also accumulated in Gxs aragonite microcosms, but the contribution to overall dissimilarity is much lower than that in Gmq spring. Besides, Caldimicrobium was a bacterial genus enriched in Gxs aragonite microcosms, in contrast to in-situ water and silicate microcosms, whereas Candidatus Kryptobacter and Thermus were more abundant in silicate microcosms. The differences in microbial accumulations among different mineral types in the same spring implied that mineral chemistry may exert extra deterministic selective pressure in drawing certain species from the bulk water communities, in addition to stochastic absorption on mineral surface. Taken together, our results highlight the special niche partitioning determined by mineralogical compositions and further confirm that minerals could be used as “fishing bait” to enrich certain rare microbial species.

## 1. Introduction

It is well known that microbial species are coupled and co-evolve with the environment where they live [1,2]. Hot springs, remarkably similar to ancient environments, are one of the optimum systems to study the direct influence and feedback between Earth materials (e.g., geochemical environment) and microbial populations [1]. The terrestrial hot springs are characterized by extreme geochemical conditions and diverse mineralogical composition [3]. These geothermal features usually harbor tremendous diversity of uncultivated or unexplored microbial ‘dark matter’ [4], with novel metabolic capacities and special adaption strategies [5,6,7,8]. A growing body of evidence suggests that temperature, pH, and geochemistry conditions shape microbial distribution and functional changes in geothermal ecosystems [9,10,11,12,13,14,15,16,17] and some studies recognize the microbial effect on mineral precipitation and weathering [18,19]. Thermodynamics-based and bioenergetics/growth yields-based methods showed that both energy supplies and demands have effects on microbial population growth in geothermal ecosystems [20,21]. Thermophiles can obtain energy from minerals [22]. A recent study revealed that archaea and bacteria could form their own guilds and occupied individual niches in hot springs by using network analysis [16]. However, this finding mainly focused on the role of water geochemistry (i.e., pH and temperature) on niche partitioning, little is known whether minerals could provide a special niche that is different from those determined by water geochemistry in hot springs. The effect of mineralogical compositions on niche partitioning is important because community composition, metabolic function distributions, and community assembly processes are heavily influenced by mineral composition [3,19,23,24]. Network-based analysis has shown its advantage in deciphering ecologically meaningful interactions between microbial species and between microbes and environmental conditions [1,23,24,25]. This offers a new approach to study the contribution of mineralogical composition to the ecological niche partitioning of thermophiles when considering water geochemistry together with mineralogical composition.

The various properties of minerals may lead to distinct microbial species colonizing on their surface, such as differences in nutrition supply, redox status, and chemical compositions of the minerals [26,27,28,29]. For example, bacteria preferentially colonized on minerals with Fe and P [30,31]. A similar selective effect has also been observed in fungal and archaeal communities. For instance, the fungal Glomeromycota appeared to preferentially associate with ferrihydrite surfaces, compared with kaolinite and quartz [32]. Besides, Thaumarchaeota (Marine Group I) were detected on apatite surfaces because phosphorus is one of the main variables determining Thaumarchaeota niches [28,33]. On the other hand, the special metabolic ability and the tolerance capacities of some microbial species help them colonize on certain mineral surfaces. For instance, phylum Chytridiomycota has been found to be abundant on oligoclase with high Al concentrations due to their tolerance of Al^3+^ [28,34]. Burkholderia was the key genus responsible for apatite weathering [35] due to its ability to solubilize phosphorous or mobilize iron [36]. Furthermore, ecological processes underlying microbial community assembly were highly dependent on mineral types and thus resulted in distinctly different community compositions [32]. A recent study highlighted the important role of the heterogeneous selection effect on microbial community in hot spring sediments [37]. However, there is little experimental data to show whether specific minerals or mineral compositions in hot spring sediments could selectively enrich specific microbes or communities.

Tengchong geothermal field, located in the southwest of Yunnan Province, is one of the most active geothermal areas in China [38,39]. We have gained much knowledge about (hyper)thermophiles and water geochemistry in these habitats [10,11,40]. In this study, we first employed the SparCC approach to investigate potential interactions among microbial species, water geochemistry, and mineralogical composition in Tengchong hot springs. Then, given the fact that Tengchong geothermal regions mainly have two types of sediment contexts, namely silicate deposits (e.g., Gumingquan in Rehai geothermal region) and carbonate deposits (e.g., Gongxiaoshe in Ruidian geothermal region), we conducted in-situ mineral microcosm experiments with four minerals (aragonite with 100–250 μm and 250–500 μm particle sizes, albite, K-feldspar, and quartz) in silicate-dominated alkaline spring Gumingquan and carbonate-dominated neutral hot spring Gongxiaoshe for 70 days. We compared the changes in microbial community composition and diversity between bulk water and mineral cultures by using high-throughput sequencing of 16S rRNA genes. The objectives of this study are: (i) to reveal if sediment mineralogical compositions in terrestrial hot springs offer a special ecological niche that is different from water geochemistry via network analysis, and (ii) to determine if various types of minerals selectively favor certain species from the source community in bulk water.

## 2. Materials and Methods

### 2.1. The Hot Spring Datasets Used for Co-Occurrence Network Construction

The Rehai and Ruidian are two major geothermal fields in Tengchong. Numerous hot springs with distinct temperature, pH, geochemical, and sedimentary characteristics exist in the field [37,40]. To achieve our first objective, a co-occurrence network was constructed based on the data from our previous publications [11,40]. The sediment microbial compositions, water geochemistry, and mineralogical compositions of nine springs (4 from acid springs and 5 from neutral-alkaline springs) were obtained from our previous study (Table S1) [11]. Most of the geothermal types occurring in the study area are included to obtain an overall picture of niche partitioning of thermophiles determined by both water geochemistry and mineralogical compositions. In these nine springs, the temperature ranged from 53 to 92.1 °C and the pH from 2.81 to 9.4. The concentrations of Na^+^, K^+^, and Cl^−^ elevated in neutral-alkaline springs, whereas Fe^2+^, total Fe, SO_4_^2−^, NH_4_^+^, and TN were increased in the acid springs. The pyrosequencing data of 16S rRNA gene were obtained through 454 GS FLX platform and processed with QIIME. A more detailed data processing procedure can be found in our previous publications [11,40].

### 2.2. Co-Occurrence Network Construction and Statistical Analysis

To reveal the niche partitioning of thermophiles regarding mineralogical compositions and water geochemistry, a co-occurrence network was built by using SparCC algorithm [41,42]. Top 100 OTUs in abundance were selected to build the microbial network. Only significantly correlated relationships were included in the network with SparCC correlation strength >0.5 and *p* < 0.05. The network was visualized using Gephi (v0.9.2) [43]. Node-based properties including degree, closeness centrality, and betweenness centrality were calculated. A node with high degree (locally important), high betweenness centrality (globally important), and high closeness centrality (central position) indicates that it is an influential node for both the whole network and the local cluster and it is located at the central position of the whole network. Network properties including average clustering coefficient, average path length and modularity were measured. The definitions and calculations of these indices were described previously [23]. Briefly, the average clustering coefficient describes the degree to which nodes tend to cluster together and the average path length represents average network distance between all pairs of nodes. Generally, modularity >0.4 indicates that the network has a modular structure and could be separated into several individual modules. A module represents a group of species within the same taxonomic affiliations or sharing a similar ecological niche, highly connecting with each other inside modules. Thus, the module could be used to investigate whether water geochemistry and minerals generate individual niches for microbial species and how they interact with each other. The edges in the network could be either positive or negative. Positive connections indicate similar favorable environmental conditions, niche overlapping, and syntrophism, whereas negative connections imply competition, niche partitioning, or antagonism.

### 2.3. Hot Spring Selection and Mineral Microcosms Design

**Figure 1:**
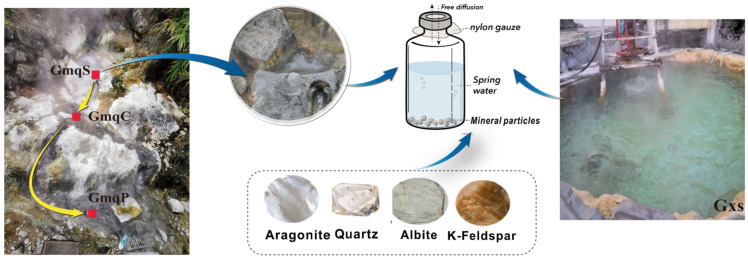
Design of the in-situ cultivation experiment. Four fresh minerals including aragonite, quartz, albite, and K-feldspar were separately filled in serum bottles and cultivated in Gumingquan source (GmqS) and Gongxiaoshe (Gxs) spring.

Hot springs Gumignquan and Gongxiaoshe (shorted as Gmq and GXS, respectively) were selected for mineral microcosm experiments to ascertain the role of minerals on selective enrichment of certain species. Gmq is a high-temperature and alkaline spring in the Rehai geothermal field with a short water flow path (~3 m between source and pool, shortened as GmqS and GmqP, respectively) but high velocity (Figure 1). There is a temperature gradient (from 93 °C decreasing to 82.5 °C) along the water flow direction, whereas the bulk pH (~9.3) and conductivity (~4.0) remain unchanged. The water in this spring is rich in sodium and chloride. Silicates and quartz dominated Gmq sedimentary mineralogical compositions, but slight differences in mineralogical composition were detected along this flow path (Table 1). In comparison, Gxs, located in the Ruidian geothermal field, is a neutral calcium-carbonate spring [39], with intermittent gushing water from the middle of the octagon-shaped pool. Carbonates such as calcite and aragonite dominated Gxs sedimentary mineralogical compositions with well-developed sinter. Spring water at bottom in Gmq and GXS were anaerobic to microaerobic as previously reported [11].

Four types of minerals, including aragonite, quartz, K-feldspar, and albite, were chosen to do mineral microcosm experiments (Figure 1). Minerals were obtained from https://www.bzwz.com/ accessed on January 2011, under product numbers of UKSAK, SARM49, GBW03116, and GBW03134, respectively. Aragonite minerals were ground to sequentially pass three different mesh sizes (500 μm, 250 μm and 100 μm), and the particle sizes of 250–500 μm (aragonite_500) and 100–250 μm (aragonite_250) was retained. Other minerals were prepared in one size of 100–250 μm and all these minerals were then washed by sonication in distilled deionized water to remove ultrafine particles [30]. The alkaline spring Gmq and neutral spring Gxs were chosen to perform in-situ microcosms due to the following reasons: (i) Gmq and Gxs represent two distinct geothermal features given the mineralogical compositions, namely silicate-hosted (pH > 9.3) and carbonate-hosted (pH > 7.3) springs, but with similar microbial communities in water(Figure 1); (ii) sediment in GmqC (channel of Gmq) contained abundant Desulfurococcales belonging to Crenarchaeota, distinctly different those in GmqS and GmqP (Figure 1) [11,40]. The contents of carbonate minerals (mainly aragonite) differentiate GmqC sediment from the GmqS and GmqP sediments (Table 1). These comparisons triggered us to do mineral microcosm experiments to explore if certain mineral leads to the enrichment of certain species.

In the mineral microcosm experiments, about 2 g of mineral particles were put into each sterilized 5 mL glass serum bottle and filled with spring water directly collected from the vent for Gmq and from the middle of the pool for Gxs (Figure 1). The bottles were sealed with high-temperature resistant nylon gauze (100-μm pore size) and aluminum caps to avoid the potential mineral or rock contamination from surroundings, but allowing free diffusion of microbes and chemicals in spring water between the inside and the outside of the bottles (Figure 1). These mineral-containing serum bottles were incubated completely submerged in the water at GmqS and Gxs for 70 days from June 23rd to August 31st, 2014. At the end of the incubation, the planktonic microbial cells in the in-situ bulk water were also collected by filtering through 0.22 μm pore size syringe filter (Pall Corporation, Port Washington, NY, USA). The bottles and filters were frozen and transported in dry-ice, and stored at −80 °C in the laboratory until further microbial community analysis.

### 2.4. DNA Extraction, PCR Amplification, and Sequencing

Triplicates of DNAs in bulk water (collected on filters) and sediments (~0.5 g), as well as in minerals slurry (~0.5 g) were extracted by using FastDNA SPIN Kit for Soil (MP Biomedical, OH, USA) according to the manufacturer’s protocol. The extracted triplicates of DNAs were pooled and amplified using a universal primer set 515F (5′-GTGYCAGCMGCCGCGGTAA-3′)–806R (5′-GGACTACHVGGGTWTCTAAT-3′) as previously described [40]. Unique 8-bp barcodes were added at the 5′-end of reverse primers to identify each sample.

Three technical triplicates were done for PCR and each 25-μL PCR reaction system contained 0.25 μL Ex Taq polymerase (Takara, Dalian, China), 2.5 μL of 10× PCR buffer, 2 μL of template DNA (~10–15 ng), 0.5 μL of 10 mmol/L dNTP and 1.0 μL of 10 mmol/L of each primer and 17.75 μL sterilized ddH_2_O. The amplification procedure was as follows: an initial denaturation at 95 °C for 5 min, followed by 30 cycles of 30 s denaturation at 94 °C, annealing at 54 °C for 30 s and 1 min extension at 72 °C. Then a final extension was added at 72 °C for 10 min [40]. The amplified products for the same sample were pooled and checked by gel electrophoresis. DNA-containing gels were purified by using the Qiagen gel extraction kit (Qiagen, Valencia, CA, USA). Ultimately, purified amplicons of all samples were pooled in equal quantity for Illumina Miseq sequencing.

### 2.5. Data Processing and Statistical Analysis

Pair-end raw sequences for mineral microcosms were joined with fastq-join program [44], and then assigned to different samples according to barcodes by using split_libraries_fastq.py in QIIME [45]. Simultaneously, low quality sequences were removed by allowing no barcode errors and average quality scores higher than 30. The chimeric sequences were checked by using UCHIME [46]. After removing the low-quality and chimeric sequences, operational taxonomic units were clustered at a 97% sequence identity level by using an UPARSE algorithm [47]. Taxonomy was assigned using the ribosome database project (RDP) classifier algorithm [48] with both the Greengene and Silva reference databases to ensure more accurate and finer phylogenetic affiliations. The phylum “Bacteroidetes” from the Silva database actually is equal to the “FCB group”, so the term “FCB group” was used instead. The phyla in this supergroup were shown in Figure S1. The OTU table was normalized by random resampling to 30,000 sequences. Then, species richness (Hill number, q = 0), taxonomic diversity (Hill number, q = 1) and Faith’s phylogenetic diversity (PD) were calculated. Hierarchical clustering was performed for in-situ water and mineral microcosms with a paired group algorithm based on Bray–Curtis dissimilarity to group samples with a similar assemblage composition. To determine the percentage of contribution of individual genera to the overall dissimilarity between paired clusters identified in the Hierarchical clustering tree, a similarity percentage analysis (SIMPER) [49] was performed using Bray–Curtis dissimilarity. Clustering and SIMPER were done in PAST 2.12 [50].

## 3. Results

### 3.1. Highly Modular Structure Revealed by Microbial Network Analysis

**Figure 2:**
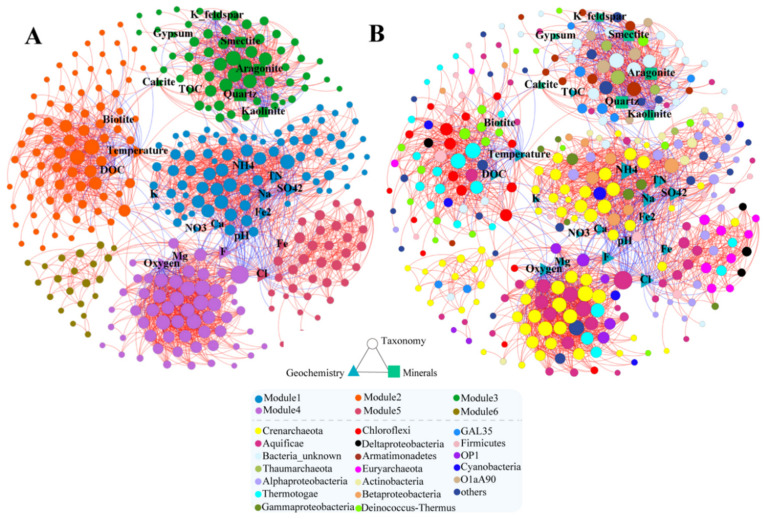
The co-occurrence networks among taxa, geochemistry, and sedimentary mineralogical compositions in the Tengchong hot springs. (**A**) The network with nodes clustered into six distinct groups. (**B**) The same network with nodes colored by taxonomic affiliations at phylum level (class for Proteobacteria). Blue and red edges indicate negative and positive interactions between nodes, respectively. The size of nodes is proportional to the number of degrees.

In this study, to identify the contribution of water geochemistry and mineralogical composition to nonrandom co-occurrence patterns in hot springs, a molecular ecological network was constructed based on the SparCC correlation between microbial phylotypes, geochemistry parameters, and sediment mineralogical composition. The global properties for this network were further calculated. The co-occurrence network included 352 nodes and 3007 edges, with an average clustering coefficient of 0.656 and an average path length of 3.444. The relatively higher modularity index of 0.725 indicated the co-occurrence patterns could be separated into individual modules [51,52]. Indeed, 6 modules were detected (Figure 2 and Table 2). Each module has its closely related abiotic factors (Table 2), except for the module 6, which was dominated by Crenarchaeota and no abiotic factor was involved (Figure 2B). As previously reported, a linear correlation could be detected in some of these abiotic factors. For example, pH was negatively correlated with Fe^2+^, total Fe and SO_4_^2−^, but positively correlated with Na^+^, Cl^−^ and F^−^.

The majority of the OTUs in the network were affiliated to Crenarchaetoa (17.87%), Aquificae (10.95%), Alphaproteobacteria (6.05%), Thermotogae (5.76%), and Thermi (5.48%) (Figure 2B). A list of keynodes was identified via some characteristics of network topological properties (Table S2).

**Figure 3:**
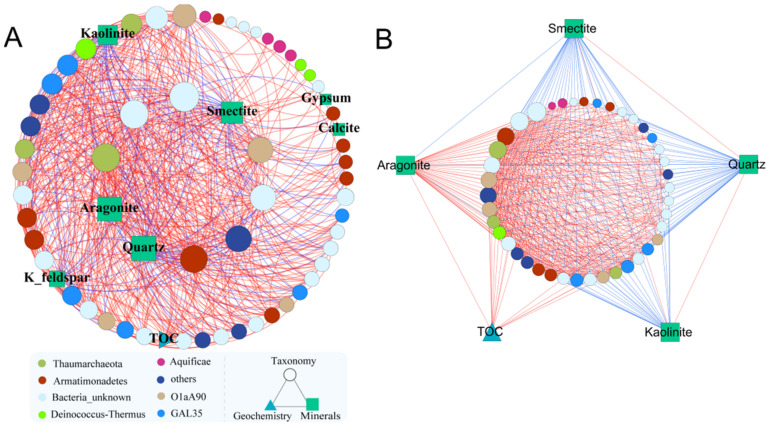
The sub-networks highlighting the influence of the minerals on species interactions. (**A**) This subnetwork represents the module 3 extracted from Figure 2. The nodes in the inner circle were among the top 10 nodes ranked by degree. Nodes were arranged counter-clock-wise for inner and outer circles, respectively. (**B**) A subnetwork for aragonite and its first neighbors in module 3. The size of nodes is proportional to the number of degrees. Blue and red edges indicate negative and positive interactions between nodes, respectively. Different colors for circular nodes represent different taxa at the phylum level (class for Proteobacteria).

In the mineralogical composition-dominated module (Figure 3), clay minerals (kaolinite, smectite), carbonate (aragonite, calcite), gypsum, and other silicate minerals (K-feldspar, quartz) affected the microbial interactions. Aragonite, quartz, and smectite were among the highest node degrees (Figure 3A and Table S3), but they exert distinct contributions to the subnetwork (Figure 3B and Table S3). Specifically, quartz and smectite were negatively connected with other nodes, whereas aragonite was mainly positively associated with other nodes (Table S3). In addition, the unknown bacteria made up a higher proportion (31.75%) in this sub-network. Overall, network analyses provide a new dimension to document niche partitioning in hot springs and highlight the role of mineralogical compositions in determining microbial interaction dynamics.

### 3.2. Microbial Community Composition in Gmq In-Situ Water and Mineral Microcosms

**Figure 4:**
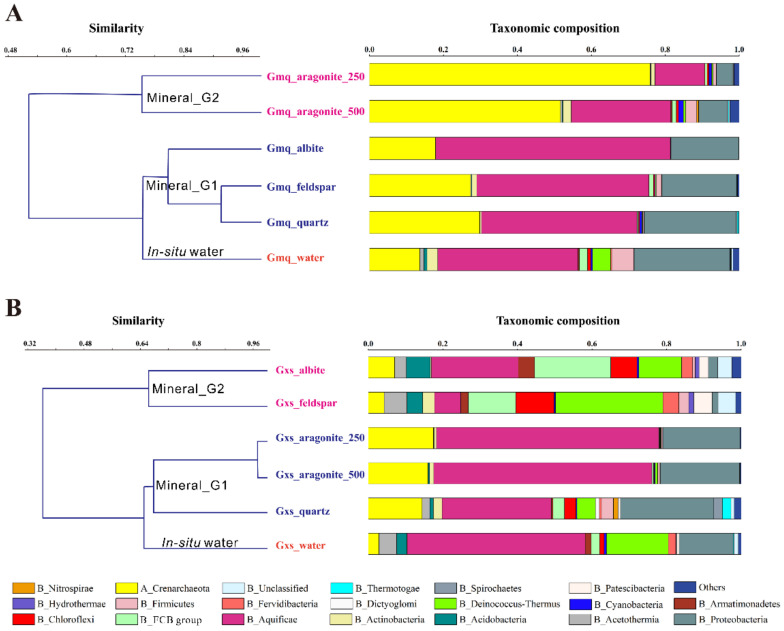
Clusters of community compositions for in-situ water and mineral microcosms in Gmq (**A**) and Gxs (**B**) springs. Clustering the dominant phyla was performed with paired group algorithm based on Bray–Curtis dissimilarity. The sub-clusters separated in the clustering trees were identified as “In-situ water”, “Mineral_G1” and “Mineral_G2” groups. “others” category indicates rarer taxa with relative abundance <1% in each sample.

**Figure 5:**
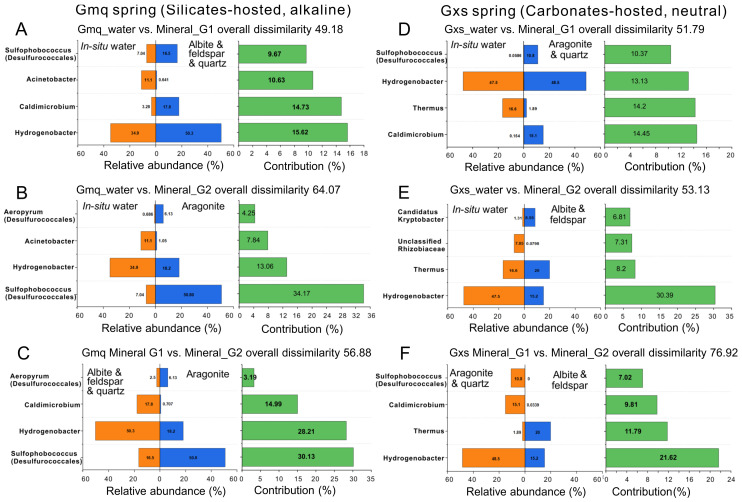
Taxonomic genera that contributed to up to 50% of the overall dissimilarities between paired groups in the Gmq and Gxs springs. (**A**) Gmq_water versus Mineral_G1 (Albite, K-feldspar, and quartz); (**B**) Gmq_water versus Mineral_G2 (aragonite_250 and aragonite_500); (**C**) Mineral_G1 versus Mineral_G2 in the Gmq spring; (**D**) Gxs_water versus Mineral_G1 (aragonite_250, aragonite_500 and quartz); (**E**) Gxs_water versus Mineral_G2 (Albite and K-feldspar); (**F**) Mineral_G1 versus Mineral_G2 in the Gxs spring.

Clustering results showed that microbial communities in aragonite microcosms (Gmq Mineral_G2) differed from those in Gmq in-situ water and silicates and quartz microcosms (Gmq Mineral_G1) (Figure 4A). Gmq in-situ water was dominated by Aquificae (~37.9%), followed by Proteobacteria (~25.9%), Crenarchaeota (~13.6%), Firmicutes (~5.97%), and Deinococcus-Thermus (~4.9%) (Figure 4A). Similar to water, Aquificae, Proteobacteria, and Crenarchaeota were the most dominant phyla in the albite, K-feldspar, and quartz microcosms (Figure 4A), and the total proportion of these three phyla reached to 94% of the entire community. However, substantial changes were observed in the aragonite microcosms, with the abundances of Crenarchaeota intensively increasing (Figure 4A), in particular the genera Sulfophobococcus and Aeropyrum within Desulfurococcales (Figure 5B,C). The less abundant taxa in aragonite microcosms included Aquificae, Proteobacteria, Firmicutes, and Actinobacteria (Figure 4A). Furthermore, the Desulfurococcales abundance was higher in the aragonite_250 microcosm than that in the aragonite_500 microcosm (68.3% vs. 37.8%). The shared and unique OTUs among in-situ water and mineral microcosms were shown in a sample-OTUs bipartite network (Figure S2).

The SIMPER analysis showed that 409 genera contributed to dissimilarity between water and aragonite bacterial communities. The top four major genera were listed for contributing to over 50% of the overall dissimilarity between paired groups (Figure 5A–C). Gmq Mineral_G1 group (albite, K-feldspar, and quartz) slightly enriched Sulfophobococcus (within Desulfurococcales) [53] and Caldimicrobium (within Thermodesulfobacteriales) [54] and Hydrogenobacter (within Aquificales) in compared to Gmq in-situ water (Figure 5A). Gmq Mineral_G2 group (aragonite_250 and aragonite_500) abundantly accumulated Sulfophobococcus, followed by Aeropyrum (within Desulfurococcales) [55] as compared to both in-situ water (Figure 5B) and Mineral_G1 (Figure 5C).

### 3.3. Microbial Community Composition in Gxs In-Situ Water and Mineral Microcosms

Gxs in-situ water community was closer to aragonite and quartz microcosms (Mineral_G1), much different from those in silicate microcosms (Mineral_G2) (Figure 4B). The main taxonomical entities of in-situ water were the bacterial phyla Aquificae (~47.7%), Deinococcus-Thermus (~16.6%), Proteobacteria (~14.5%), and Acetothermia (~4.74%). Similar community compositions were found in the quartz microcosms, dominated by Aquificae (~29.3%), Proteobacteria (~25.04%), Crenarchaeota (~14.4%), and Deinococcus-Thermus (~5.0%). Compared to the Gmq, the aragonite had weak selective effect for certain species in this carbonate-hosted neutral hot spring Gxs. The aragonite microcosms were dominated by Aquificae (~49.1%), Proteobacteria (~22.4%), Crenarchaeota (~16%), and Deinococcus-Thermus (~1.9%). The microbial communities colonizing on the albite and K-feldspar surfaces were significantly different from in-situ water. The albite surfaces were dominated by Aquificae (~23.4%), Bacteroidetes (~20.4%), Deinococcus-Thermus (~11.5%), and Crenarchaeota (~7.1%). Yet, the K-feldspar surfaces were largely dominated by Deinococcus-Thermus (~28.8%), followed by Bacteroidetes (~12.7%), Chloroflexi (~10.3%), and Aquificae (~7.3%).

These results were further supported by the SIMPER analysis (Figure 5D–F), since the most dissimilar types were Mineral_G1 (aragonite and quartz) and Mineral_G2 (albite and K-feldspar) (overall dissimilarity = 76.92), followed by water and Mineral_G2 (albite and K-feldspar) (overall dissimilarity = 53.13). The SIMPER analysis indicated that Caldimicrobium, Thermus, Hydrogenobacter, and Sulfophobococcus were the greatest contributors to dissimilarity between in-situ water and Mineral_G1, and Mineral_G1 markedly enriched Caldimicrobium and Sulfophobococcus (Figure 5D). Besides, Hydrogenobacter, Thermus, unclassified Rhizobiaceae, and Candidatus Kryptobacter [56] were the top four genera, which contributed to the differences between in-situ water and Mineral_G2, accounting for 30.39%, 8.2%, 7.31%, and 6.81%, respectively (Figure 5E). Hydrogenobacter, Thermus, Caldimicrobium, and Sulfophobococcus were the top four genera which contributed to the differences between Mineral_G1 and Mineral_G2, accounting for 21.62%, 11.79%, 9.81%, and 7.02%, respectively (Figure 5F). Candidatus Kryptobacter, and Thermus were accumulated in albite and K-feldspar microcosms in comparison to those in in-situ water (Figure 5E) and aragonite microcosms (Figure 5F).

## 4. Discussion

By integrating microbial data with accompanied metadata (water geochemistry and sedimentary mineralogical composition) via network analysis, we could better understand how external abiotic factors influence the microbial community assembly [57]. Specifically, the formation of different modules within an ecological network indicates the niche partitioning and/or synergistic relationships [58]. In our results, a highly modular structure was detected (Figure 2). Previous studies emphasized that water geochemistry (i.e., pH and temperature) played an important role in niche differentiation in hot springs [14,16]. Adding to this, the highly modular structure in the co-occurrence patterns revealed that sediment mineralogical composition could offer special ecological niche that are different from those determined by water geochemical parameters. Furthermore, a high proportion of unclassified bacteria within mineral-dominated module were detected, possibly indicating that minerals could be a potential matrix used to enrich microbial dark matter in hot springs. Indeed, our results showed that mineral microcosms accumulated rare (hyper)thermophilic species from bulk water communities (Figure 5). From the microbial diversity aspect, close relationship between minerals and unclassified microbes suggest minerals could promote microbial diversification since minerals and biology have co-evolved [2] and our results confirmed that mineral-microbe interaction create novel niche, which agree well with the theory of Diversity Begets Diversity (DBD) model which states biodiversity promotes further diversification when species interactions create novel niches [59].

The relative importance of nodes for the network could be identified by some network-based or node-based properties. For instance, nodes that are important for clusters probably have high degree and low betweenness centrality, and those important for the whole network may have high degree and high betweenness centrality [60,61]. Furthermore, high closeness centrality means nodes are located at the central position in the network. Accordingly, the OTU affiliated to Hydrogenobacter (closest match with Hydrogenobacter T-8 [4]) has the highest degree, betweenness centrality, and closeness centrality values (Table S2), implying that the central role of this taxon in stabilizing the microbial network structure. The relatively high degree and low betweenness of aragonite may indicate that it is an influential node in the mineral-dominated module 3 (Table S2 and Figure 3). Furthermore, the abovementioned Hydrogenobacter was negatively correlated with Fe^2+^, SO_4_^2−^, and NH_4_^+^, whereas positively correlated with pH, Na^+^, F^−^ and Cl^−^ in the network, in consistent with previous studies revealing that Hydrogenobacter mainly existed in neutral-alkaline hot springs with relatively low concentrations of Fe^2+^, SO_4_^2−^, and NH_4_^+^ [62] and high concentrations of Na^+^, F^−^, and Cl^−^ [10,11,41].

Mineral types and background environments both influence the microbial species colonized on certain minerals, as revealed by the SIMPER and clustering results (Figure 4 and Figure 5). Smaller dissimilarities were found in community compositions between aragonite microcosms and bulk water in Gxs where carbonate dominated rather than that between silicate microcosms and bulk water in Gmq, where silicate dominated. Niche conservation is one possible reason for such a phenomenon. As documented, niches for microorganism are conservative, so that species are prone to occupy similar environmental conditions in new geographical ranges or time periods [63]. In our case, microbial species dwelling in Gxs spring were well-adapted to carbonate-rich conditions and thus aragonite could not exert external selective pressure on microbial species in this habitat. Similarly, the opportunity for each microbe to occupy the niches provided by silicates is roughly equal in Gmq; therefore, similar communities were observed in the silicates and quartz microcosms as compared to bulk water. In general, stochastic absorption on mineral surface in flowing water could happen, but great differences were observed among different mineral types for the same spring, which implied that minerals may exert extra deterministic selective pressure in drawing certain species from the bulk water communities in addition to random absorption.

Some studies found that in terrestrial hot springs, greater microbial diversities in the sediments than those in water [40,64]. According to our results, different mineral types favored different species may ensure the high microbial diversity in hot spring sediments, given hot spring sediments are highly heterogeneous in mineralogical composition. Previous studies have reported that minerals may provide advantageous surface properties or microhabitats to protect microbes against extrinsic harsh environmental conditions [65,66]. For example, acidic microenvironments are often extensively developed at the sulfide-mineral surfaces in the mining waste rock drainage, which may support the growth of acidophilic bacteria and further facilitate sulfide-mineral oxidation [67,68]. Although carbonate could not buffer pH in the alkaline condition, we indeed found that Gmq water pH (>9.3) exceeds the growth pH range for the abundantly accumulated Desulfurococcales (mainly Sulfophobococcus (pH 6.5–8.5) [53]) (Figure 5). The existence of filaments (i.e., Sulfophobococcus) or pilus-like appendages (i.e., Aeropyrum) may be responsible for adherence of the cells on mineral surface. Furthermore, their fast growth ensured that they could prevail when they arrived at the mineral surface, which is a survival strategy for mineral-associated microbes [32]. Then the generation of cell aggregates may help these microbes overcome the unfavorable alkaline conditions. Besides, since these microbes are heterotrophic, the porous structure of aragonite is beneficial for the organic carbon absorption, as evidenced by the positive association between total organic carbon (TOC) in sediments and aragonite (Figure 3B). Our previous study also showed that dissolved organic carbon (DOC) in Tengchong hot spring waters were much higher in June and August than in January, which was thought to be the major contributor for the temporal variation of microbial communities in these waters [11].

Mineral microcosms may influence the availability of oxygen and ion strength for microbial species. The closest match in Sulfophobococcus is Sulfophobococcus zilligii, which was reported to be strictly anaerobic and grow at really low salinity (<0.2%) [53], whereas Aeropyrum, taking Aeropyrum pernix for example, is strictly aerobic and prefers a much higher salinity strength (1.8 to 7.0%) [55]. The Aeropyrum species in the hot spring may be one of low-salinity adapted relatives, and the mineral aragonite may provide refuges with some salinity strength. According to our measurement, the dissolved oxygen of the surface water in Gmq was extremely low (9–15 μg/L in August) due to the high temperature and high velocity, but we found strictly aerobic Aeropyrum co-existed with strictly anaerobic Sulfophobococcus in Gmq aragonite microcosms. Taken together, the co-existence of genera Sulfophobococcus and Aeropyrum (belonging to Desulfurococcales) within aragonite microcosms in Gmq could be spatially separated due to their distinctly different requirements for oxygen and ion strength. This could be achieved due to the more porous structure of aragonite [69] than silicates such as albite [70]. This could be another reason to explain the community differences between different minerals.

The microbial species simultaneously accumulated in certain mineral microcosms, which may also imply the dependent relationship between them. For example, we observed Candidatus Kryptobacter accumulated in albite and K-feldspar microcosms; this genus was proposed to have a heterotrophic lifestyle with the putative capacity for iron respiration, and the silicates such as albite and K-feldspar contain a small amount of iron, which may favor growth of this genus. Besides, genomic evidence showed that this genus had conspicuous nutritional deficiencies, thus likely leading to a partnership with other microbes, such Armatimonadetes lineages and Thermus spp. [56] In our results, the accumulation of Armatimonadetes and Thermus also occurred in albite and K-feldspar. A previous study showed that Armatimonadetes played major roles in adhesion to surfaces and biofilm formation due to the capability of ‘functional amyloids’ secretion. Candidatus Kryptobacter may be responsible for remodeling and digestion of this extracellular matrix. Furthermore, Candidatus Kryptobacter encodes a nitrous oxide reductase (reduce nitrous oxide to dinitrogen), which cooperated with Thermus (reduce nitrate to nitrous oxide) to complement the denitrification pathway in hot springs [56]. These finding suggest minerals may serve as “baits” to fish the rare and undiscovered species in the hot springs.

## 5. Conclusions

This study demonstrated that mineralogical compositions could create special ecological niches that are distinguishable from those determined by water geochemistry via network analysis in Tengchong hot springs. In addition, the in-situ microcosm experiments with four minerals (aragonite, albite, K-feldspar, and quartz) further showed that minerals favor certain species from the bulk water communities, but the intensity of the selective effect on microbial community varied with mineral types and bulk environments (silicate-hosted vs. carbonate-hosted). Specifically, aragonite microcosms in Gmq spring enriched archaeal genera Sulfophobococcus and Aeropyrum in Desulfurococcales as compared to both in-situ water and silicate microcosms, largely due to their mobility and fast growing, as well as the porous structure of aragonite. Weak enrichment of Sulfophobococcus was also observed in Gxs aragonite microcosms. Besides, bacterial genus Caldimicrobium was another genus enriched in Gxs aragonite microcosms, in contrast to in-situ water and silicate microcosms, whereas Candidatus Kryptobacter and Thermus were more abundant in silicate microcosms than in-situ water and aragonite microcosms and the partnership between these two lineages has been proposed. Collectively, our findings emphasize the importance of mineralogical composition in regulating microbial community assembly and in selectively enriching microbial species, and expand the current understanding of the factors determining niche partitioning in hot springs. Future work should focus on the feasibility of using different mineralogical compositions to selectively enrich rare and uncultured species in hot springs and other aquatic settings.

## Figures and Tables

**Table 1:** The physicochemical parameters and main mineralogical composition in Gumingquan and Gongxiaoshe springs.

Spring Name	Location	Spring ID	Conductivity mS/cm	pH	Temperature °C	Main Minerals
	Source	GmqS	4.0	9.35	93	quartz, feldspar
Gumaguma	Channel	GmqC	4.0	9.36	89	quartz, aragonite, goethite
	Pool	GmqP	3.9	9.30	82.5	quartz, feldspar
Gongxiaoshe		Gxs		7.29	73.8	aragonite, calcite

**Table 2:** Information for individual modules.

Module No.	Node	Intra-Module Edges		Inter-Module Edges		Related Abiotic Factors
		Positive	Negative	Positive	Negative	
1	88	663	53	61	81	pH, K^+^, Ca^2+^, Na^+^, TN, NH_4_^+^, SO_4_^2−^, NO_3_^−^, Fe^2+^
2	79	430	49	21	7	Temperature, DOC, Biotite
3	63	445	117	18	11	TOC, Kaolinite, Smectite, Calcite, Aragonite, Quartz, Gypsum, K-feldspar
4	59	680	57	32	64	Oxygen, Mg, F^−^
5	37	237	15	47	23	Total Fe, Cl^−^
6	21	62	0	9	0	---

## Data Availability

Raw sequencing data were included in Short Read Archive database at NCBI under the BioProject accession numbers PRJNA178165 and PRJNA703944.

## Supplementary Materials

The following are available online at https://www.mdpi.com/article/10.3390/microorganisms9051020/s1, Figure S1: The phyla belonging to the “FCB” group. The summarizations of sequences were extracted from the normalized OTU table at 30,000, Figure S2: Sample-OTU bipartite graph showing the shared and unique OTUs for in-situ water samples and mineral microcosms in Gmq and Gxs springs, Table S1: Water geochemistry and mineralogical compositions of the nine springs involved in the co-occurrence network analysis, Table S2: Top 20 nodes ranked by node degree, betweenness centrality and closeness centrality, Table S3: Comparison of networks topological characteristics in sedimentary mineralogical composition.
