# Supplementary material for: Minerals Determined a Special Ecological Niche and Selectively Enriched Microbial Species from Bulk Water Communities in Hot Springs
Source: Microorganisms. 2021 May 10;9(5):1020. doi: 10.3390/microorganisms9051020 (PMC8151621; doi:10.3390/microorganisms9051020)
Supplement: Supplementary file 1 [file microorganisms-09-01020-s001.zip › microorganisms-1191327-supplementary.pdf]

Minerals Determined a Special Ecological Niche and Selectively Enriched Microbial Species from Bulk Water Communities in Hot Springs

Fangru Li [1,†], Shang Wang [2,†], Qing He [2], Wenhui Zhang [1], Dongyi Guo [1], Yidi Zhang [1], Wanming Hai [1], Yuxuan Sun [1], Hailiang Dong [1] and Weiguo Hou [1,*]

[1] State Key Laboratory of Biogeology and Environmental Geology, China University of Geosciences,
Beijing 100083, China; fangruli@cugb.edu.cn (F.L.); 2157190005@cugb.edu.cn (W.Z.); dongyiguo@cugb.edu.cn (D.G.); 2157180007@cugb.edu.cn (Y.Z.); 2157180004@cugb.edu.cn (W.H.); 2157180006@cugb.edu.cn (Y.S.); dongh@cugb.edu.cn (H.D.)

[2] CAS Key Laboratory of Environmental Biotechnology, Research Center for Eco-Environmental Sciences, Chinese Academy of Sciences, Beijing 100085, China; shangwang@rcees.ac.cn (S.W.); qinghe_st@rcees.ac.cn (Q.H.)

* Correspondence: weiguohou@cugb.edu.cn

† These authors contributed equally to this work.

1 Supplementary Tables

Table S1. Water geochemistry and mineralogical compositions of the nine springs involved in the co-occurrence network analysis.

	Drty-1	Drty-2	Drty-3	Zzq	GmqP	Gxs	JmqR	Jz	SrbzD
pH	3.2	2.81	3.09	4.7	9.4	7.72	8.98	7.04	8.27
Temperature (℃)	87.8	66.25	53	92.1	83.5	75	84.7	80.7	72.1
TOC (%)	0.4	1.6	0.8	0.6	0.4	3.7	0.6	4.5	1.1
DOC (mg/l)	117.59	7.38	7.4	7.4	6.37	83.18	10.6	6.5	1.63
TN (mg/l)	28.8	3.83	3.8	3.8	0.3	0.39	0.3	0.23	0.26
NH_4^+ (mg/l)	1309.52	166.67	47.62	500	6.67	4.29	4.52	7.38	4.76
Fe^{2+} (mg/l)	203.11	412.19	370.37	3.46	0	0	0.18	0	0
Oxygen (mg/l)	16.35	19.49	21.88	24.47	7.86	30.21	7.23	19.16	16.45
Mg (mg/l)	4.9	4.1	0.62	0.5	0	4.3	0	4.2	0
Total Fe (mg/l)	9.7	21.5	23.8	0.5	0	0	0	0	0
Ca^{2+} (mg/l)	82	63.8	22.83	25.7	23.3	36.6	22.9	45.4	24.3
K^+ (mg/l)	176.9	62.2	89.23	37.6	215.3	120.5	203.6	115.5	126.8
Na^+ (mg/l)	13.3	28.7	34.69	103.1	1461	832	1386.3	774	880.7
Cl^- (mg/l)	19.3	8.8	4.55	38.2	506.5	146.2	543.2	137.7	323
SO_4^{2-} (mg/l)	713.4	1380.5	594.23	91.8	18.8	21.8	22.4	23	29.1
F^- (mg/l)	1.6	1.5	1.41	1.4	12.7	6.6	13.7	6.3	8
NO_3^- (mg/l)	7	2.7	0	1.6	0.3	0	0.4	1.3	1.2
Quartz (%)	52.7	44.7	44.9	9.4	23.8	0	14.6	20.7	27.8
K-feldspar (%)	3.8	9.7	5.7	46.2	38.7	0	39.9	19.7	26.9
Calcite (%)	1.4	1.7	0.6	0	2.5	12.1	3.4	12.3	0
Aragonite (%)	0	0	0	2.9	0	86.3	0	0.6	0
Gypsum (%)	2.4	1.6	1.3	2.9	1.8	0	2	3.6	0.7
Kaolinite (%)	9.5	14.9	30.9	11.2	5	0	7.5	7.8	10.7
Smectite (%)	8.4	14.5	8.3	20.2	17.9	0	17.4	26.1	14.3
Biotite (%)	5.5	3.6	0	3.3	4.1	0	4.9	2.4	0

Table S2. Top 20 nodes ranked by node degree, betweenness centrality and closeness centrality

Id	taxonomy	degree	betweenness centrality	closeness centrality
otu1979	Hydrogenobacter	65	4827.002493	0.404206
NH4+	env	59	3010.919342	0.38964
otu3353	Desulfurococcaceae_unclassified	50	2031.650432	0.390079
otu3325	Fervidobacteriumnodosum	50	2147.474295	0.335597
otu469	Thermodesulfobacteriaceae_unclassified	49	537.462655	0.342574
otu2416	Aquificaceae_unclassified	49	714.528001	0.358178
otu2325	Bacteria_unknown	48	633.443935	0.308929
otu1592	Thermotogaceae_unclassified	48	1463.172808	0.3343
otu2330	Aquificaceae_unclassified	47	322.800412	0.351269
otu33	Thermoproteaceae_unclassified	46	490.501308	0.328273
otu1431	Ralstonia	46	1088.30584	0.380638
Quartz	mineral	46	4777.005543	0.350202
otu1424	Desulfurococcaceae_unclassified	45	224.099105	0.341223
otu1093	Bacteria_unknown	45	892.015336	0.299567
Aragonite	mineral	44	455.085113	0.299308
otu1094	Candidatus Nitrosocaldus	44	529.722188	0.298533
otu1081	Thermoprotei_unclassified	44	645.981269	0.340887
Na	env	44	3674.690478	0.402794
Cl	env	44	6532.739977	0.420925
SO42-	env	43	4294.995379	0.405627

Table S3. Comparison of networks topological characteristics in sedimentary mineralogical composition

factors	degree	closeness centrality	betweenness centrality	clustering
Quartz	46	0.350505	4266.451383	0.380676
Aragonite	44	0.301215	373.819471	0.445032
Smectite	37	0.29811	212.558493	0.524024
Kaolinite	31	0.351215	3588.203474	0.466667
K_feldspar	21	0.294567	164.163046	0.704762
Calcite	9	0.348744	3082.537373	0.388889
Gypsum	5	0.275616	2.016511	0.8
Biotite	3	0.300693	73.150532	0

2 Supplementary Figures

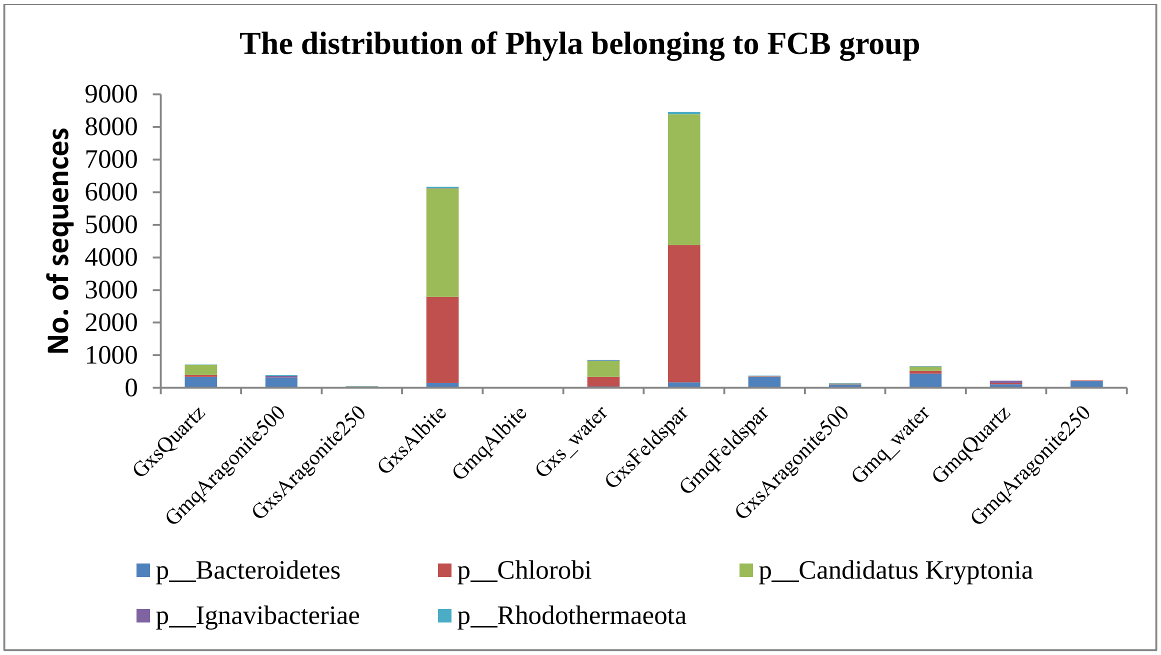

Figure S1. The phyla belonging to the "FCB" group. The summarizations of sequences were extracted from the normalized OTU table at 30000.

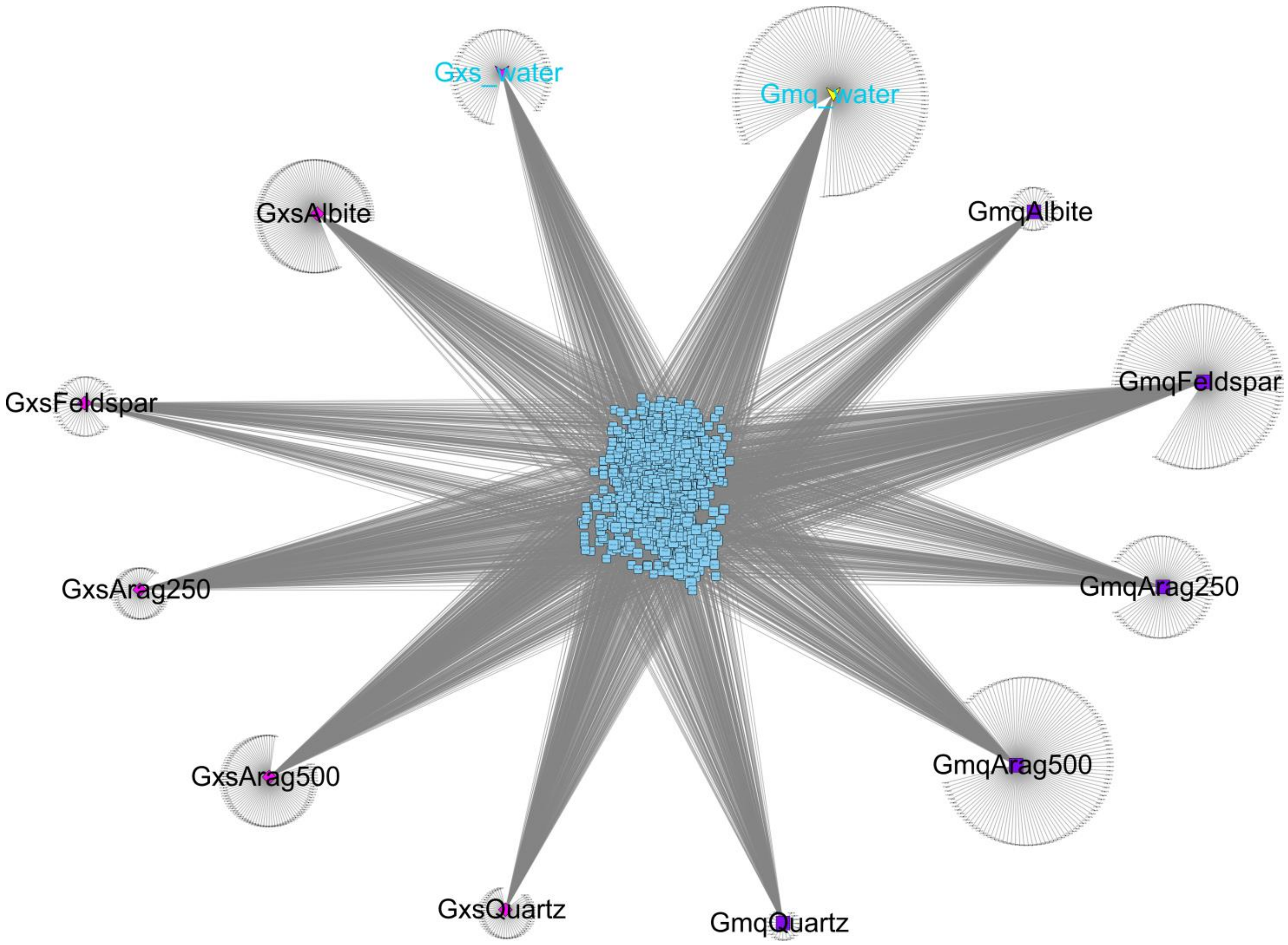

Figure S2. Sample-OTU bipartite graph showing the shared and unique OTUs for in-situ water samples and mineral microcosms in Gmq and Gxs springs.
